# IL-17 and IL-22 are pivotal cytokines to delay wound healing of *S. aureus and P. aeruginosa* infected skin

**DOI:** 10.3389/fimmu.2022.984016

**Published:** 2022-10-07

**Authors:** Jean-Claude Lecron, Sandrine Charreau, Jean-François Jégou, Nadjet Salhi, Isabelle Petit-Paris, Emmanuel Guignouard, Christophe Burucoa, Laure Favot-Laforge, Charles Bodet, Anne Barra, Vincent Huguier, Jiad Mcheik, Laure Dumoutier, Julien Garnier, François-Xavier Bernard, Bernhard Ryffel, Franck Morel

**Affiliations:** ^1^ Laboratoire Inflammation, Tissus Epithéliaux et Cytokines, UR15560, Université de Poitiers, Poitiers, France; ^2^ Laboratoire Immunologie et Inflammation, Centre Hospitalier et Universitaire (CHU) de Poitiers, Poitiers, France; ^3^ Qima-Bioalternatives (Qima Life Sciences), Gençay, France; ^4^ Laboratoire de Bactériologie, Centre Hospitalier et Universitaire (CHU) de Poitiers, Poitiers, France; ^5^ Service de Chirurgie Plastique, Centre Hospitalier et Universitaire (CHU) de Poitiers, Poitiers, France; ^6^ Service de Chirurgie Pédiatrique, Centre Hospitalier et Universitaire CHU) de Poitiers, Poitiers, France; ^7^ De Duve Institute, Université catholique de Louvain, Brussels, Belgium; ^8^ Laboratoire d'Immunologie et Neurogénétique Expérimentales et Moléculaire (INEM) - Unité Mixte de Recherche (UMR) 7355, Centre National de la Recherche Scientifique (CNRS) et Université d’Orléans, Orléans, France

**Keywords:** skin, wound healing, cytokine, Th17, pathogen, inflammation

## Abstract

**Introduction:**

Although the presence of pathogens in skin wounds is known to delay the wound healing process, the mechanisms underlying this delay remain poorly understood. In the present study, we have investigated the regulatory role of proinflammatory cytokines on the healing kinetics of infected wounds.

**Methods:**

We have developed a mouse model of cutaneous wound healing, with or without wound inoculation with *Staphylococcus aureus* and *Pseudomonas aeruginosa*, two major pathogens involved in cutaneous wound bacterial infections.

**Results:**

Aseptic excision in C57BL/6 mouse skin induced early expression of IL-1β, TNFα and Oncostatin M (OSM), without detectable expression of IL-22 and IL-17A/F. *S. aureus* and *P. aeruginosa* wound inoculation not only increased the expression of IL-1β and OSM, but also induced a strong cutaneous expression of IL-22, IL-17A and IL-17F, along with an increased number of infiltrating IL-17A and/or IL-22-producing γδ T cells. The same cytokine expression pattern was observed in infected human skin wounds. When compared to uninfected wounds, mouse skin infection delayed the wound healing process. Injection of IL-1α, TNFα, OSM, IL-22 and IL-17 together in the wound edges induced delayed wound healing similar to that induced by the bacterial infection. Wound healing experiments in infected Rag2KO mice (deficient in lymphocytes) showed a wound healing kinetic similar to uninfected Rag2KO mice or WT mice. Rag2KO infected-skin lesions expressed lower levels of IL-17 and IL-22 than WT, suggesting that the expression of these cytokines is mainly dependent on γδ T cells in this model. Wound healing was not delayed in infected IL-17R/IL-22KO, comparable to uninfected control mice. Injection of recombinant IL-22 and IL-17 in infected wound edges of Rag2KO mice re-establish the delayed kinetic of wound healing, as in infected WT mice.

**Conclusion:**

These results demonstrate the synergistic and specific effects of IL-22 and IL-17 induced by bacterial infection delay the wound healing process, regardless of the presence of bacteria *per se*. Therefore, these cytokines play an unexpected role in delayed skin wound healing.

## Introduction

The wound healing process is the final step to restore tissue integrity following the acute inflammation induced by a skin injury, when a chronic inflammatory step is not engaged. The mechanisms and controls of wound healing (proliferation, migration, extracellular matrix protein synthesis, angiogenesis…) are complex and involve collaborative efforts of many cell types (fibroblasts, keratinocytes, immune cells…) and numerous soluble factors (inflammatory mediators, growth factors, cytokines, antimicrobial peptides) produced at the lesion site (1). These mechanisms also depend on the environment of the wound, including the presence of germs capable of using skin wound as a gateway to colonize the body. These germs can modify the inflammatory response, the immune response and the healing process.

It is a common belief that wound bacterial infection is detrimental to wound healing, especially by delaying the process. Beside commensal skin microbiota regulating homeostasis of skin host immune response (2), wound infections can disrupt the host-bacteria equilibrium favoring bacterial growth toward altered wound healing processes. These processes depend on host immune response, bacterial count, species and virulence of bacteria, as well as the synergistic effect of the different species (3). The gram-positive bacteria *Staphylococcus aureus (S. aureus)* is a major pathogen of numerous skin infections such as cellulitis, impetigo or folliculitis that can be complicated by septicemia, whereas the gram-negative bacteria *Pseudomonas aeruginosa (P. aeruginosa*) is responsible for infection of chronic wounds associated with delayed wound healing and cutaneous starting point-nosocomial infections (4, 5). *P. aeruginosa* and *S. aureus* are the two most common causes of chronic wound infection. These both species are frequently found together in wounds and may exhibit synergistic interactions (6–10). Wound infection is a serious public health concern and could originate from operative procedures on the skin during surgery, acute trauma during accidents or chronic pressure ulcers in the context of diabetes.

Wound healing resolution is a medical and surgical challenge, especially in the case of skin infection, whereas wound healing delay or inhibition is a critical concern. The situation is nevertheless more complex since paradoxically, it is known to surgeons that an infected wound should not be closed, and that, if necessary, it be reopened to prevent abscess and bacterial dissemination, summarized about 40 years ago by the plastic surgeon Raymond Villain’s famous aphorism “Paix sur la plaie aux germes de bonne volonté” (11).

However, the mechanisms slowing down the wound healing kinetic during infection remain poorly understood, especially if it is caused by bacteria or bacterial derived products intra se, or due to host immune response and the inflammatory mediators released. For about 15 years, the production and role of growth factors and proinflammatory cytokines over skin inflammatory processes have been extensively documented (12), albeit their precise role on the last phase of the inflammatory process –i.e., wound healing- remains partially known (13). Recent studies report potential involvement of IL-17 and/or IL-22 cytokinesin this process, with a delicate balance to secure the beneficial and deleterious effects of these cytokines (14).

By studying wound healing regulation by cinnamaldehyde, Ferro et al. reported that *P. aeruginosa* delayed wound healing and induced IL-17 expression (4). In agreement, exogenous IL-17 led to delayed wound closure (15), whereas this phenomenon is abolished in IL-17A KO mice or with IL-17 blocking mAbs (15, 16). In contrast, it has been suggested that IL-22 could be a promising therapeutic agent for epithelial repair (17). In a model of type II diabetic db/db mice, systemic administration of IL-22-fc (a fusion protein that prolongs cytokine half-life *in vivo*) accelerated wound healing in S aureus infected wounds (18).

By studying the role of cytokines on cutaneous physiology and physiopathology, we and others have shown that the pro-inflammatory cytokines IL-22, IL-17A and Oncostatin M (OSM) are produced in inflammatory skin and that they target keratinocytes and fibroblasts. They induce the production of antimicrobial peptides (AMP), cytokines and chemokines, promote cell proliferation and migration and inhibit keratinocyte differentiation (19–24). In combination with TNFα and IL-1β, which are also reported to target keratinocytes, IL-22, IL-17A and OSM (M5 cocktail of cytokines) have a powerful synergistic effect on these functions (21, 23). Given their properties, in the present study we have investigated the expression of these cytokines during skin wound healing, and questioned their ability to modulate the process.

By using a mouse model of excisional cutaneous wounds co-infected or not with *S. aureus* and *P. aeruginosa*, we show that the infection specifically increases the expression of IL-17A, IL-17F and IL-22, and that these cytokines are responsible for the delayed wound healing observed in infected wounds. We discuss the beneficial effects of an acceleration or slowing down of the healing kinetics in view of developing new therapeutic approaches to skin infections and scarring.

## Materials and methods

### Bacterial strains and culture

Cultures of *S. aureus* (ATCC 29213) and *P. aeruginosa* (ATCC 27853) were prepared by inoculating Mueller-Hinton agar growth medium (Biorad) and incubated overnight at 37°C. For wound inoculation, a bacterial mixture containing both strains in equal proportions was prepared in 1X PBS (Dulbecco) by resuspending each strain at a final density of 2.5 x10^8^ CFU/ml. Bacterial concentration was determined by measuring the absorbance at 600 nm using an Ultrospec-10 spectrophotometer (Amersham Biosciences) and by colony-forming unit (CFU) counts for each strain controlled by serial 10-fold dilutions of the bacterial suspensions and plating on either MH (Mueller Hinton)/vancomycin and CNA (Columbia Naladixic Acid) selective agar plates.

### Animals

Eight to ten-week-old C57BL/6J mice were purchased from Janvier Labs (Le Genest, France). Mice were acclimatized for at least 1 week before experiments. Rag2KO, IL-17R and IL-17R/IL-22KO mice were provided by Marc Le Bert (INEM, CNRS, Orléans, France). IL-22KO mice were from L Dumoutier (de Duve Institute, Belgium) All transgenic mice and their WT littermates were bred and housed in the animal facility of Poitiers University (Prebios), under specific pathogen-free conditions and maintained on a 12 h light/dark cycle, with food and water ad libitum. Protocols and animals were approved by the regional ethics committee for animal experimentation (COMETHEA-CE86) under the agreement number CE-2012-21.

### Excisional skin wounding, bacterial infections and measurement of wound areas

After anesthesia by an intraperitoneal injection of a xylazine (10 mg/ml)/ketamine (1 mg/ml) solution (100 μl of this solution/10 g of animal weight), the back skin of mice was shaved and remaining hairs were removed using a depilatory cream (Veet@, Reckitt Benckiser, France). Four excisional wounds were performed by puncturing the skin with a 4 mm-round punch (Stiefel) and removing the skin biopsy. Twenty microliters of the bacterial suspension (5X10^6^ CFU) or the PBS control solution were directly applied on each wound. After spreading of the bacterial mixture on the wound, mice were housed individually to avoid grooming behavior of infected wounds.

To follow wound healing kinetics, the surface of each wound was measured daily using Image J software on pictures of back skin of mice including a meter stick as reference. The wound area was determined in mm².

To analyze the effect of cytokines on wound healing kinetics, three injections of 10 µl of carrier-free recombinant cytokine solution diluted in 1X PBS at a final concentration of 25 µg/ml (IL-17A, IL-17F, OSM, TNFα, IL-22 and IL-1α; all from R&D systems, Europe) or combinations were performed in each wound edges. Control mice received PBS injections.

For RNA isolation, each wound biopsy was collected using a 6 mm punch (Stiefel) after mouse euthanasia, rapidly snap-frozen and stored in liquid nitrogen. For flow cytometry analysis, the whole skin containing the four excisional wounds (representing an 8 cm² surface) was collected five days after excision and wound infection, in RPMI medium supplemented with 10% of Fetal Calf Serum (FCS), 1% penicillin/streptomycin (P/S) solution and 200 ng/ml of gentamycin (all products were purchased from Invitrogen) and stored at 4°C until cell dissociation. For bacterial analysis, the 6 mm punch was dissociated in 0.5ml PBS and cell count was performed as described above in bacterial strains and culture.

### Patients

This study included adult patients presenting uninfected (n=12) or infected wounds (n=7). Skin biopsies were obtained during surgical treatment of the wound. For uninfected wounds, sampling was performed from surgical edge wounds without pathological healing process. For infected wounds, edge sampling was performed during their surgical trimming before antibiotherapy. A swab was sampled for culture bacteriological analysis in the Microbiology Laboratory of the Poitiers Hospital. Two patient wounds were infected by three germs (hemolytic *Streptococcus-Proteus mirabilis-Corynebacterium spp and S. epidermitis- Propionobacterium acnes*-*S. aureus*) and the other ones were infected with *S. aureus*. Normal skin samples were obtained from surgical samples of abdominoplasty or breast reduction surgery and were used as controls (n=16). The biopsies were immediately frozen in liquid nitrogen before RNA extraction.

All of our studies involving human tissues were approved by the Institutional Ethics Committee on Human Experimentation (Comité de Protection des Personnes Ouest III) of the Poitou-Charentes Region. This study was conducted according to the Declaration of Helsinki principles, and oral informed consent was obtained from participants before inclusion.

### RNA isolation and real-time quantitative RT-PCR

Skin total RNA was isolated and reverse transcribed as previously described (21). Quantitative real time PCR was carried using the LightCycler-FastStart DNA MasterPlus SYBR^®^ Green I kit on LightCycler 480 (Roche Diagnostics, Meylan, France). The reaction components were 1X DNA Master Mix, and 0.5 µM of sense and anti-sense oligonucleotides purchased from Eurogentec (Eurogentec France, Angers, France), designed using Primer3 software. Samples were normalized to three independent control housekeeping genes (G3PDH, RPL13A and ACTB for human samples and G3PDH, HMBS and B2M for mouse samples) and reported according to the ΔΔCT method as RNA fold increase: 2ΔΔCT= 2ΔCT sample- ΔCT reference.

### Cell preparation and flow cytometry

Collected skin biopsies comprising the four excisional wounds were cut into small pieces using scissors and scalpel before overnight treatment in a 2.5 U/ml solution of dispase diluted in complete RPMI at 4°C. Then, skin tissues were incubated in an enzymatic solution composed of 50 µg/mL of Liberase TM Research Grade (Roche Diagnostics, Basel, Switzerland), 25 ng/ml of collagenase I and 100 µg/mL of DNase I (Sigma-Aldrich, St. Louis, MO) for 4 hours at 37°C before filtration through a 100 µm cell strainer followed by a second filtration through a 40 µm cell strainer and two washes in complete RPMI by centrifugation at 1700 rpm for 10 min. After the last centrifugation, cell pellets were resuspended with 250 µl of StemPro Accutase solution (Gibco) for 2 min at 37°C. After washing, cells were resuspended in complete RPMI and stimulated for 4 hours in the presence of phorbol myristate acetate (PMA, 50 ng/ml), ionomycin (750 ng/ml) and Golgi Plug (1 µl for/10^6^ cells; BD Biosciences). Before extracellular staining, cells were incubated with Fc Block (BD Biosciences) for 10 minutes and then for 15 minutes at 4°C with the following antibodies: V500-conjugated anti-CD45, BV421-conjugated anti-CD3ϵ, FITC-conjugated anti-TCRγδ (all from BD Biosciences) and Zombie NIR for cell viability (BioLegend, San Diego,CA). For intracellular staining, cells were permeabilized with the Cytofix/Cytoperm kit and labelled for 15 min at 4°C with PE-conjugated anti-IL-17A (BD Biosciences) and PercP-eFluor710-conjugated anti-IL-22 (eBioscience) antibodies. Data were collected on a FACS Verse instrument (BD Bioscience) and analyzed using FlowJo software.

### Statistical analysis

Statistical analysis of significance was calculated using either the Mann-Whitney U-test or the Kruskal Wallis one-way analysis of variance by ranks followed by a Dunn’s post-test. p values of 0.05 or less were considered as significant, and all data are represented as mean +/- SEM. Comparison study used the Spearman rank correlation test.

## Results

### *S. aureus* and *P. aeruginosa* inoculated in skin excisional wounds delay the wound healing process

**Figure 1:**
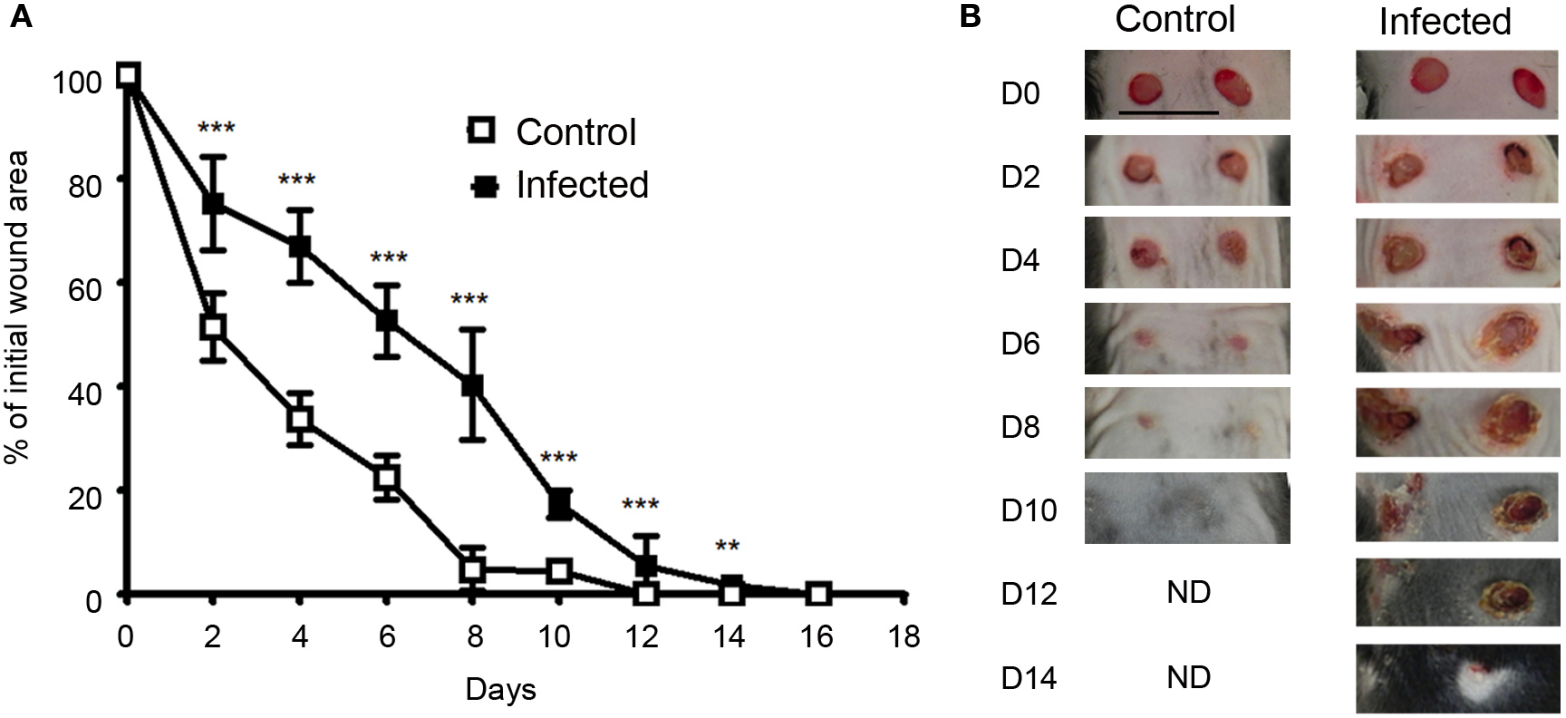
Wound closure is delayed in infected skin excisional wounds.**(A)**Time course of wound closure expressed as the percentage of initial wound area at each time point, in the control wound (□) or wound infected with a mixture of *S. aureus* and *P. aeruginosa* (■). The data are expressed as the mean +/- SEM of 5 independent experiments, with at least 4 mice/group; ** *p*<0.01, ***p<0.001. **(B)** Representative photographs of the control and infected wounds at days 0, 2, 4, 6, 8, 10, 12 and 14 after injury..

The kinetics of macroscopic wound closure was studied after performing 4 mm excisional wounds on the back skin of mice. By measuring the surface of the lesions every 2 days, we observed a progressive closure with nearly complete healing on day 8 for control mice/uninfected wounds. In contrast, when wounds were co-infected with a mixture of *S. aureus* and *P. aeruginosa*, the wound closure was almost achieved at day 12, with significant differences with uninfected wound from day 2 to day 14 (**Figures 1A, B**).

### IL-17 and IL-22 expression in infected wound

**Figure 2:**
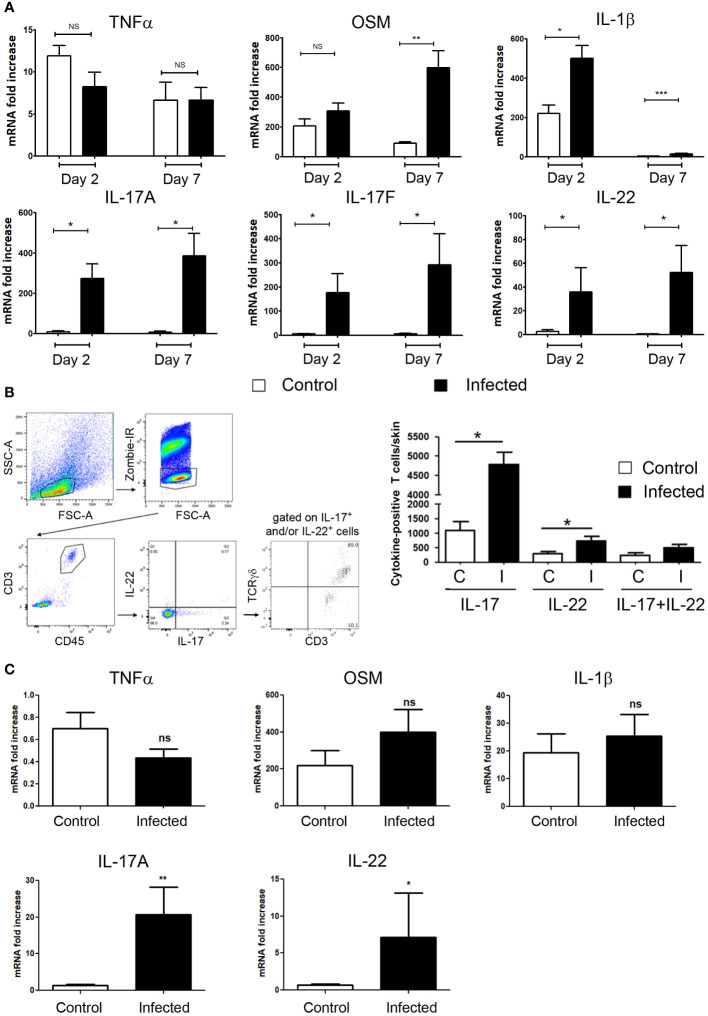
IL-17 and IL-22 are produced by T cells in infected skin excisional wounds. **(A)** Cytokine mRNA expression into control or *S. aureus* and *P. aeruginosa* infected wounds were determined by RT-qPCR at day 2 and day 7. Each bar represents the mRNA fold increase over normal skin, in control-wounded skin (white) or infected wounded skin (black). The data correspond to the mean +/- SEM of 4 independent experiments; * *p*<0.05, ** *p*<0.01, ***p<0.001; **(B)** Five days after wound infection by the bacterial mixture (infected, I) or application of a control PBS solution (uninfected, C), an 8 cm^2^ biopsy of back skin including 4 excisional wounds was collected and underwent mechanical and enzymatic treatment for cell dissociation before analysis by flow cytometry. (B, left) Representative gating strategy to analyze IL-17 and IL-22 expressions in CD3-positive T cells and TCRγδ expression among this IL-17^+^ and/or IL-22^+^, CD3^+^ T cell population. (B, right) Quantitative analysis of the number of IL-17-, IL-22- or IL-17/IL-22-positive T cells infiltrating infected or non-infected wounded skins. Data (mean +/- SEM) are representative of one out from three independent experiments including 5 mice per group. *p < 0.05. **(C)** IL-17A, IL-22, OSM, IL-1β and TNFα mRNA levels were determined by quantitative PCR in human wounds. Each bar represents the mRNA fold increase over normal human skin in uninfected (white) or infected wounded skin (black). The data correspond to the mean +/- SEM of 12 uninfected and 7 infected skin wound samples; * *p*<0.05, ** *p*<0.01, ***p<0.001. ns, not significant..

It is well-documented that different sets of proinflammatory cytokines are produced in injured skin, whatever the etiology. With multiple target cells in skin, these cytokines have pleiotropic properties, such as not only to amplify and coordinate the inflammatory process, but also to have the ability to control it. We focused in the present study on the IL-22, IL-17, OSM, TNFα and IL-1β proinflammatory cytokines, which are produced in inflammatory human and murine skin and drive an inflammatory keratinocyte state. Taken together, their combination (M5 mixture) had a powerful synergistic biological effect on epidermal cells, stronger than other cytokine combinations studied (21, 23). Skin excision in aseptic condition induces the early skin expression of IL-1β, TNFα and OSM at day 2 and day 7, without detectable expression of IL-22, IL-17A and IL-17F (**Figure 2A**). In contrast, *S. aureus* and *P. aeruginosa* wound infection induced a strong expression of IL-22, IL-17A and IL-17F on day 2, which remained elevated on day 7. Under these conditions of infection, TNFα expression remained unchanged when compared to uninfected wound, while OSM increased about 5 times at day 7, and IL-1β enhanced at day 2 and day 7 (**Figure 2A**). As IL-22, IL-17A and IL-17F are mainly produced by T cells, we isolated CD45/CD3 immune cells from the skin of infected and non-infected mice collected at day 5 and analyzed cytokine expression by flow cytometry. This approach allows us to validate the mRNA cytokine expression pattern at the protein level and to precise the type of producing cells. We observed a significant increase in the number of IL-17A- and IL-22-producing T cells in the skin of infected mice compared to uninfected mice, while the slightly increased number of IL-17A/IL-22 double positive cells in the skin of infected mice was not significant (**Figure 2B**). These IL-17 and/or IL-22 positive CD3 T cells were 89.9% TCRγδ (**Figure 2B**).

Finally, we observed a close expression pattern in infected human skin wounds. Whereas IL-1β and OSM were similarly overexpressed in uninfected and infected wounds compared to normal skin, IL-17A and IL-22 expressions were enhanced only in infected wounds. In contrast to mice, TNFα levels were unchanged in wounds when compared to normal skin (**Figure 2C**).

### IL-17 and IL-22 induced by bacterial infection have a key role in the delayed wound healing process

**Figure 3:**
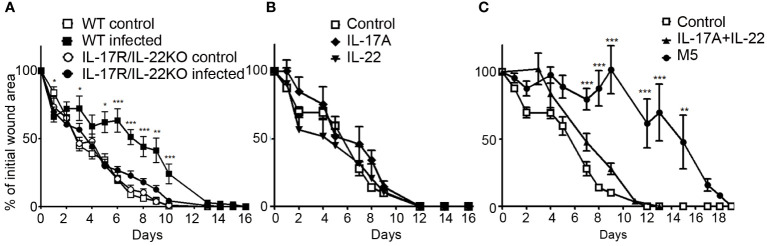
Combination of IL-17 and IL-22 are required for delayed closure in an infected skin wound. Time course of wound closure expressed as the percentage of initial wound area at each time point, **(A)** in the uninfected or infected wounds of WT mice (control □; infected ■) or IL-17R/IL-22KO mice (control ○; infected ●). **(B)** in the uninfected wound of WT mice injected with IL-17A (♦), IL-22 (▼). **(C)** in the uninfected wound of WT mice injected with IL-17A and IL-22 (▲), mix of IL-17A, OSM, TNFα, IL-22 and IL-1α (●) or PBS as a control (□). The data are expressed as the mean +/- SEM of 5 independent experiments, with at least 4 mice/group. * *p*<0.05, ** *p*<0.01, ***p<0.001..

Since *S. aureus* and *P. aeruginosa* inoculation delayed skin wound healing and specifically induced IL-17 and IL-22 expression, we wondered if these cytokines could be responsible for the delayed healing. We studied the wound healing process in IL-17RKO or in IL-22KO infected mice and observed no significant difference with wound closure time course of infected WT mice (**Supplementary Figure 1**). In contrast, the wound healing process in infected IL-17R/IL-22 double knock-out mice (IL-17R/IL-22KO) is reduced and the wound closure time course similar to that of non-infected WT mice, highlighting the key role of the combination of IL-17 and IL-22 in this process (**Figure 3A**). To confirm their key roles, IL-17A/IL-17F and IL-22 alone or in combination were injected in four sites of each wound edge after skin excision, in the absence of bacteria. As indicated in **Figure 3B**, neither IL-17A/IL-17F nor IL-22 alone was able to modify the wound closure time course compared to the control group, whereas the combination of both cytokines weakly slowed down the healing process (**Figure 3C**), demonstrating that the simple combination of IL-17A/IL-17F and IL-22 was not sufficient to explain delayed closure of an infected wound. While TNFα, IL-1β and OSM are produced in non-infected skin lesions, IL-1β and OSM are produced much more in infected wounds; given these findings, we wondered whether sufficient amounts of cytokines were necessary to reveal and potentiate the effects of IL-22 and IL-17A/IL-17F. Indeed, the injection of IL-1α, TNFα, OSM, IL-17A, and IL-22 mixture in the edge of uninfected lesions effectively delayed the wound healing process, as has been observed in the presence of bacteria (**Figure 3C**).

### The wound healing process in Rag2KO mice is not delayed by bacterial infection

**Figure 4:**
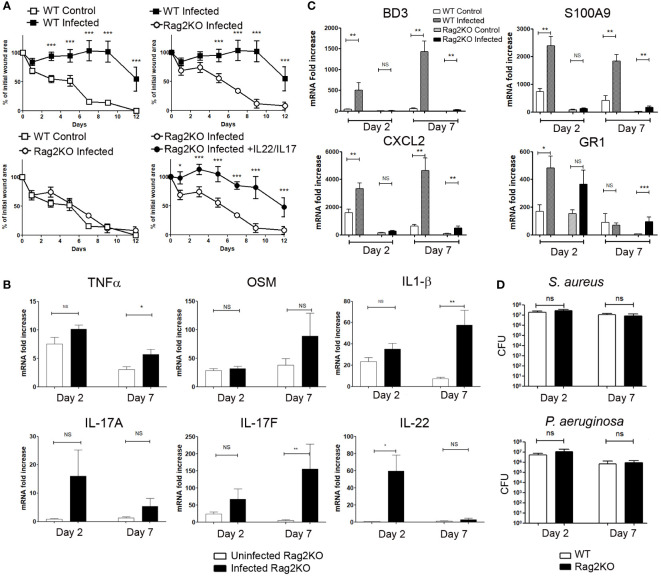
Wound closure is not delayed in infected skin excisional wounds of Rag2KO mice, and exogenous IL-17A/F and IL-22 make up for this delay. Time course of wound closure expressed as the percentage of initial wound area at each time point, **(A)** in the uninfected (□) or infected (■) wounds of WT mice, infected Rag2KO mice (○) or infected Rag2KO mice with exogenous IL-17A and IL-22 (●). The data are expressed as the mean +/- SEM of 6 independent experiments, with at least 4 mice/group; ** p<0.01, ***p<0.001. ns, not significant. **(B)** Cytokine mRNA expression into control or infected wounds of Rag2KO mice was determined by RT-qPCR at day 2 and day 7. Each bar represents the mRNA fold increase over normal skin, in control-wounded skin (white) or infected wounded skin (black). The data correspond to the mean +/- SEM of 3 independent experiments; * *p*<0.05, ** *p*<0.01, ***p<0.001. ns, not significant. **(C)** BD3, S100A9, CXCL2, GR1 mRNA expression into control or infected wounds of WT and Rag2KO mice were determined by RT-qPCR at day 2 and day 7. Each bar represents the mRNA fold increase over normal skin in control-wounded WT mice skin (white), infected wounded WT mice skin (hatched), in control-wounded Rag2KO mice skin (dotted), infected wounded Rag2KO mice skin (black). The data correspond to the mean +/- SEM of 3 independent experiments; * *p*<0.05, ** *p*<0.01, ***p<0.001. ns, not significant. **(D)** The bacterial load after inoculation in skin wounds is unchanged between WT and Rag2KO mice. CFU determination for *S. aureus* and *P. aeruginosa* in wounded WT and Rag2KO mice skin 2 and 7 days after inoculation of *S. aureus* and *P. aeruginosa*. The data correspond to the mean +/- SEM of 3 independent experiments, ns, not significant.

As IL-17A/IL-17F and IL-22 are produced mainly by T lymphocytes in infected wounds, we performed wound experiments in the presence or absence of bacteria in Rag2KO mice (deficient in mature lymphocytes). The wound healing process reported at day 7 after aseptic excisions was similar for WT and Rag2KO mice (**Supplementary Figure 2**) and wound healing achieve at day 12. In contrast to WT infected mice, the wound healing process in infected Rag2KO wounds was not delayed by infection, with a kinetics of healing comparable to that of uninfected WT wounds (**Figure 4A** and **Supplementary Figure 2**).

In uninfected Rag2KO wounds, IL-1β, TNFα and OSM mRNA levels were enhanced at days 2 and 7 when compared to uninjured Rag2KO skin (**Figure 4B**). In infected Rag2KO wounds, the levels of IL-1β, TNFα and OSM were comparable at day 2 to those of uninfected wounds, whereas their expression was enhanced at day 7 for TNFα and IL-1β (**Figure 4B**).

As for uninfected WT mice (**Figure 2A**), the expression of IL-22 and IL-17A/F was undetectable or low in uninfected Rag2KO wounds, except a discrete increase of IL-17F at day 2 when compared to normal skin (**Figure 4B**). *S. aureus* and *P. aeruginosa* wound inoculation did not significantly induce the expression of IL-17A, whereas they stimulated IL-22 on day 2 and IL-17F on day 7. OSM, IL-1β and IL-17A levels in Rag2KO infected wound remained significantly lower than in infected WT mice wounds (**Figures 2A** and **4B**). In the same conditions, although TNFα and IL-17F levels were close in the two strains of mice, IL-22 levels were strongly reduced at day 7 in Rag2KO infected wounds (**Figures 2A** and **4B**). It suggests that innate cells were also involved in the production of IL-17F and at least early for IL-22.

To further demonstrate the role of IL-22 and IL-17A/F amongst the proinflammatory cytokines able to delay wound healing, we injected exogenous recombinant IL-22 and IL-17 in the edges of infected wounds of Rag2KO mice. Whereas the healing kinetic of infected Rag2KO wounds was as fast as that of uninfected WT wounds, the combination of exogenous IL-22 and IL-17A/IL-17F delayed the wound healing process in infected Rag2KO mice at the same level as with infected WT mice (**Figure 4A**).

### Regulation of the inflammatory response by infection during wound healing process

In the course of the wound healing in Rag2KO mice compared to WT, we further evaluated markers of the inflammatory response such as the antimicrobial peptides β-defensin 3 (BD3) and S100A9, the chemokine CXCL2 or neutrophil infiltration, all of which were strongly induced by the M5 mixture (21). In WT mice, BD3 expression was significantly induced in wounds infected by *S. aureus* and *P. aeruginosa* at day 2 and to an even greater extent at day 7, but not in uninfected wounds. (**Figure 4C**). In contrast, S100A9 and CXCL2 expressions were already highly induced in uninfected wounds of WT mice at day 2 as well as day 7, and these levels were much more highly increased by infection. The same induction was observed in infected wounds of Rag2KO mice in particular at day 7, but at levels much lower than those of WT mice (**Figure 4C**). Regarding the neutrophil GR1 marker, mRNA levels increased in non-infected WT and Rag2KO wounds at day 2 (**Figure 4C**). Application of *S. aureus* and *P. aeruginosa* mixture on wounds resulted in further GR1 mRNA overexpression at day 2 for WT mice and at day 7 for Rag2KO mice (**Figure 4C**).

Lastly, we compared the impact of the time course of wound healing on the bacterial load 2 and 7 days after inoculation of skin wounds. We detected approximately 2.10^7^ *S. aureus* CFU/wound in WT mice at day 2 and 1.10^7^ at day 7, and 8.10^6^ P*. aeruginosa* CFU/wound at day 2 and 8.10^5^ at day 7. Surprisingly, we could not point out any clear-cut differences between WT and Rag2KO mice, either at day 2 or at day 7, both for *S. aureus* and *P. aeruginosa* (**Figure 4D**). Furthermore, no suggestive signs of infection were observed in wounds of mice receiving PBS as a control, and neither *S. aureus* nor *P. aeruginosa* were detected.

## Discussion

Skin wound healing is a regulated sequence of events controlled by multiple factors in view of restoring integrity, and it has been known by surgeons and nurses for over a century that this mechanism is delayed or stopped by bacterial wound contamination. In addition to the bacterial count and species, it depends on the number and virulence of the species present, and on the synergy between them. The wound healing process is also dependent on host immune response (3). However, despite numerous studies, the signals slowing down this healing of infected wounds remain partially understood. The role of soluble factors associated with inflammatory response is suggested by delayed wound healing in mice with sepsis induced by *P. aeruginosa* (25).

In this study, we reported that *S. aureus* and *P. aeruginosa* inoculated in skin excisional wounds of WT conventional mouse delay the wound healing process as reported with *P. aeruginosa* (4) and *S. aureus* (5, 26). In contrast to *S. aureus*, authors recently reported that *P. aeruginosa* did not delay healing, although the ulcers were larger than non-colonized ulcers (26). In accordance with our results in mice, the presence of different species such as Pseudomonas and Staphylococcus together correlates positively with nonhealing in humans (3). Interestingly, Canesso et al. showed that skin wound healing is accelerated in the absence of commensal microbiota in germ-free mice when compared to conventional mice, suggesting that commensal microbiota can delay the wound healing process (27).

We showed that if IL-1β, TNFα and OSM were present in uninfected excisional skin lesions, IL-17A, IL-17F and IL-22 were specifically produced after *S. aureus* and *P. aeruginosa* co-infection of the lesions. The combined action of IL-17, IL-22, IL-1β, TNFα and OSM delayed wound healing to a level comparable to the application of both *S. aureus* and *P. aeruginosa* in wounds. In accordance, numerous studies have reported the production of IL-17A, IL-17F and/or IL-22 in *S. aureus* (28–30) or *P. aeruginosa* infected mouse skins (4). These two species have the ability to promote cutaneous inflammation and to induce T cell-recruiting chemokine production (31, 32). Given that IL-1β, TNFα and OSM are mainly produced by innate immune cells and other skin resident cells, it would seem that the inflammatory process of an aseptic skin excisional lesion is primarily linked to an innate immunity process. By contrast, infection of the lesion with *S. aureus* and *P. aeruginosa* led to T cell recruitment/activation, mainly TCRγδ, and IL-17 and IL-22 production, evidencing the involvement of an additional adaptive immune response.

Finally, we observed a close expression pattern in infected human skin wounds, despite the differences between T cell skin infiltrates between mice and humans. T cell infiltrate in human skin is dominated by αβ Tcells, with less than 10% γδT cells both present in epidermis and dermis, whereas mouse skin is dominated by γδ T cells (50-70% in the dermis and more than 90% in the epidermis). In the epidermis, these γδ Tcells are represented by a majority of highly specialized cell subsets with a dendritic morphology termed DETC (dendritic epidermis T cells) with an invariant Vγ5Vδ1 TCR, not clearly evidenced in humans. Dermic γδ Tcells were mainly Vγ4 or 6. Thus, these γδ cells were characterized by a restricted TCR. Activation of γδ T cells can be performed both by innate and adaptive ligands, making these cells intermediate between innate and adaptive immunity (33). These cells have been reported to produce growth factors and to be involved in wound healing. Taking into account the differences between skin human and mouse T cells, the question of the IL-17/IL-22-producing cells in human wounded skin remains an opened question. αβ T cells, strongly present in human skin could be candidates. In *tcr*δ*^-/-^* mice, skin resident γδ DETC were replaced by αβ DETC with polyclonal αβ TCRs, as in humans (34). In humans, both αβ and γδ T cells participate to acute wound healing (35).”

The central role of IL-17 and IL-22 in delayed wound healing is underlined by the accelerated skin wound healing kinetics in infected IL-17R/IL-22KO mice when compared to infected WT mice, and it overlaps with those of uninfected WT mice. Whereas exogenous recombinant IL-17A and F and/or IL-22 injected in the edges of uninfected wounds did not modify the healing kinetic, the IL-1α-TNFα-OSM-IL-17A-IL-22 combination (the M5 combination) strongly delayed it and overlapped with those of infected mice. Since IL-1β and OSM were produced in non-infected skin lesions but much more in infected wounds, we suggest that additional exogenous cytokines are required to synergize with IL-17 and IL-22. In accordance, the M5 combination in other inflammatory skin models has a huge synergistic effect to induce *in vitro* a critical inflammatory phenotype on keratinocytes and a powerful skin inflammation *in vivo* in mice (21, 23). In a model of excisional wound, Thomay et al. reported that disruption of IL-1 signaling (IL-1RKO mice) improves the quality of skin architecture when compared to WT mice, but does not change the wound healing kinetics (36). In a model of subcutaneous infection by *S. aureus* in mice, Chan *et al.* reported that IL-17A and IL-22 have complementary roles in host defense (28). Using the same model, Cho *et al.* reported an increase of IL-17A, IL-17F, and IL-22 in the infected lesions, and that IL-17RKO mice developed lesions with increased size (30). Despite controversial data reporting pro-reparative effects for IL-17 cytokine family, most of the recent studies highlight the role of IL-17 in pathogenesis and its anti-reparative effects (review in 37). By using IL-17A KO mice and/or IL-17-blocking mAb in models of non-infected wound, two recent studies reported that IL-17A delayed wound closure (15, 16 and reviewed in 37), also reported in obese diabetic mouse (37, 38). It was suggested to target IL-17A in a near future to promote wound healing. However, Hadian et al. indicated that the studies on the involvement of IL-17 in wound healing are performed on non-infected models, and have been to be continued in infected ones, especially “since chronic wounds display increase relative abundance of *S. aureus* and *P. aeruginosa*” (39)

Regarding IL-22, and in disagreement with our data, another study reported that C57BL/6 mice deficient for IL-22R display a delay on day 14 wound healing (18). In contrast with this report, the same study indicated that IL-22-Fc promotes wound healing in db/db diabetic mice and *S. aureus* wound infected C57BL/6 mice (18). The differences could be due to different strategies; while we studied the behavior of an opened infected wound, Koluman et al. closed the wound and used dressing, which meant that two different healing conditions were to be evaluated.

In an attempt to summarize and even though these studies involved models and conditions (type of lesion, infection or not, independently studied cytokines…) different from ours, IL-17 increases skin lesion size and delays wound healing in the same way as in our results, and IL-22 appears to be beneficial to healing.

In agreement, by using Rag2KO mice, which fail to generate mature B and T lymphocytes, the healing kinetics of infected wound were faster compared to infected WT mice and overlapped with those of uninfected WT mice or infected IL-17R/IL-22KO mice. If wounds of Rag2KO mice significantly expressed IL-1α, IL-17F, TNFα and OSM, the production of IL-17Aand IL-22 was reduced and transient. This is in accordance with the prominence of γδ T lymphocytes in IL-17A, and IL-22 production in infected wounds of WT mice. In Rag2KO mice, we detected a residual production of IL-17F at day 2, suggesting that other cells such as innate immune cells are able to produce the cytokine. We suggest that ILC3 cells can participate to this production, in agreement with Li et al. reporting that dermis RORγ ILC3 produced IL-17F into wounded dermis (40).

Finally, the injection of exogenous recombinant IL-17 and IL-22 delayed the wound healing of infected Rag2KO to a similar degree as that of infected WT mice.

However, it was surprising that infected wounds of mice devoid of mature B and T cells were able to heal faster than those of infected WT mice, and we may wonder whether such processes are beneficial or detrimental. Moreover, the bacterial load of *S. aureus* and *P. aeruginosa* in the wounds of WT mice and Rag2KO mice were almost the same during the healing process, suggesting that adaptive immunity had a limited effect on this process. Regarding innate immunity, the production of BD3 and S100A9 was strongly reduced in Rag2KO wounds when compared to WT, as was that of the neutrophil-attracting chemokine CXCL2. However, the expression of the neutrophil marker GR1 was strongly enhanced in uninfected and infected wounds when compared to normal skin, and similar between infected wounds of WT and Rag2KO mice. By using a model of pneumonia induced by methicillin-resistant *S. aureus* in Rag2KO mice, Parker et al. reported that adaptive immunity is involved in lung pathology, and ascribe these deleterious effects to inflammatory cytokines (41). They described comparable levels of neutrophils in both WT and Rag2KO mice as in the present study, and that the clearance of bacteria is more effective in Rag2KO mice. It reminds, if necessary, the central role of neutrophils at least in such infectious models. In a model of colitis induced by *Citrobacter rotendium*, Vallance et al. reported that colitis, crypt hyperplasia and inflammation were attenuated in Rag1KO mice compared to WT, and suggest that adaptive immune response can be deleterious for tissues (42). In contrast to Parker et al. and the present study, bacteria were not cleared in these KO mice, but show few signs of disease. Taken together, these studies demonstrate that the adaptive immunity can also have paradoxical effects during bacterial defense, as deleterious impact on tissues, in part mediated by cytokines production by lymphocytes. Depending of the models, bacteria clearance can be delayed in lung or unaffected in lesional skin of RagKO mice.

Different reports have indicated that neutrophils are able to delay the wound healing process (43–45). This dependence of neutrophils is observed not for young mice (2-month-old) but only for older mice (6 to 20 months), whose wound healing is delayed when compared to young mice (43, 45). However, a reduced accumulation of neutrophils is observed in skin wounds of germ-free mice with accelerated wound healing compared to conventional mice (27). As we studied young mice (2.5 months), we suggest that the cytokine-delayed wound healing reported in our model could be independent of neutrophils

The ultimate goal is to evaluate the beneficial/deleterious effects of an acceleration or a slowing down of the healing kinetics in infected wounds. By studying regeneration after skin excision in the African spiny mice (Acomys), which is characterized by skin autonomy (capacity to regenerate missing skin), Seifert et al. suggested that too much inflammation would be detrimental, whereas no inflammation was not helpful either (46). We can hypothesize that in the case of acute infected wounds, slowed healing would allow a control of the infection by avoiding sepsis and would be beneficial. By contrast, in the case of chronic ulcers, we may wonder if IL-17 and IL-22 are involved in the lack of wound closure, which can consequently be deleterious. The deleterious effect of inflammatory cytokines in skin has been reported in hypertensive leg ulcer (HLU) and associated with impaired wound healing, displaying skin IL-1β and OSM hyperexpression. Furthermore, injection of exogenous IL-1β and OSM in the skin of mice mimics the skin characteristics of HLU (47).

This unexpected role of the proinflammatory cytokines IL-17 and IL-22 in control of the healing process provides new directions in control of kinetic wound healing by cytokines. Taken together, we have described new properties for IL-17 in association with IL-22 in control of skin wound healing kinetics. In inflamed tissues, it is always not one but a complex “cytokine network” that leads to a specific signature and response. In the present study, we have reported a specific cytokine signature associated with aseptic or infected wound skin, and demonstrated that IL-17 and IL-22 act synergistically with IL-1, TNFα and OSM are responsible for the delayed healing observed in infected wounds. Depending on the context -infected or not-, -acute or chronic wound inflammation- we suggest that these cytokines could be used to slow down the wound healing process, or could be blocked to accelerate the process, in view of developing new therapeutic approaches to treat skin infections and scarring. In this perspective, a recombinant human IL-22 dimer (F-652) has been designed and tested safety in phase I studies, with the purpose of controlling epithelial repair (48). We are looking forward to observing its effects on wound healing kinetics.

By extension, such approaches to modulate wound healing could facilitate and accelerate skin grafts, an emerging strategy that remains to be explored (49).

However, the precarious balance between beneficial and deleterious properties of numerous cytokines, depending on the target cells, the inflammatory and/or the infectious states, should make us more cautious before using them for beneficial adjuvant therapies.

## Data availability statement

The original contributions presented in the study are included in the article/**Supplementary Material**. Further inquiries can be directed to the corresponding author.

## Ethics statement

All of our studies involving human tissues were approved by the Institutional Ethics Committee on Human Experimentation (Comité de Protection des Personnes Ouest III) of the Poitou-Charentes Region. The patients/participants provided their written informed consent to participate in this study. Protocols and animals were approved by the regional ethics committee for animal experimentation (COMETHEA-CE86) under the agreement number CE-2012-21.

## Author contributions

J-CL, FM and F-XB contributed to conception and design of the study. EG, IPP, SC, and LFL performed the *in vivo* experiments, transcriptomic studies, and data management. NS, AB,JG and J-FJ performed and analyzed the *in vitro*, flow cytometry Experiments and data managements. CBu and CBo organize the bacterial studies and altogether wrote sections of the manuscript. VH and JM provided the human samples and wrote sections of the manuscript. FM performed the statistical analysis. BR and LD generate the transgenic mice and wrote sections of the manuscript. J-CL wrote the first draft of the manuscript. All authors contributed to manuscript revision, read, and approved the submitted version.

## Funding

This study was supported by grants from University of Poitiers, a clinical research program from Poitiers University Hospital (PHRC), CNRS and University of Orleans and European funding in Region Centre-Val de Loire (FEDER N° 2016-00110366).

## Acknowledgments

We thank Martine Garnier for her excellent technical support, Adriana Delwail (ImageUP platform, University of Poitiers) for technical help for flow cytometry and the members of the animal facility of the University of Poitiers (Prebios) and CNRS/University of Orléans. We thank Jeffrey Arsham (CHU Poitiers, France) for careful reviewing and editing the original English language manuscript. The authors thank the European Union for benefiting from its equipment grant program for research laboratories and platforms and the Poitiers University Hospital (CHU) for his grant for publication support.

## Conflict of interest

The authors declare that the research was conducted in the absence of any commercial or financial relationships that could be construed as a potential conflict of interest.

## Publisher’s note

All claims expressed in this article are solely those of the authors and do not necessarily represent those of their affiliated organizations, or those of the publisher, the editors and the reviewers. Any product that may be evaluated in this article, or claim that may be made by its manufacturer, is not guaranteed or endorsed by the publisher.
