# Supplementary material for: IL-17 and IL-22 are pivotal cytokines to delay wound healing of S. aureus and P. aeruginosa infected skin
Source: Front Immunol. 2022 Oct 7;13:984016. doi: 10.3389/fimmu.2022.984016 (PMC9585169; doi:10.3389/fimmu.2022.984016)
Supplement: Supplementary file 1 [file DataSheet_1.docx]

Supplementary Material


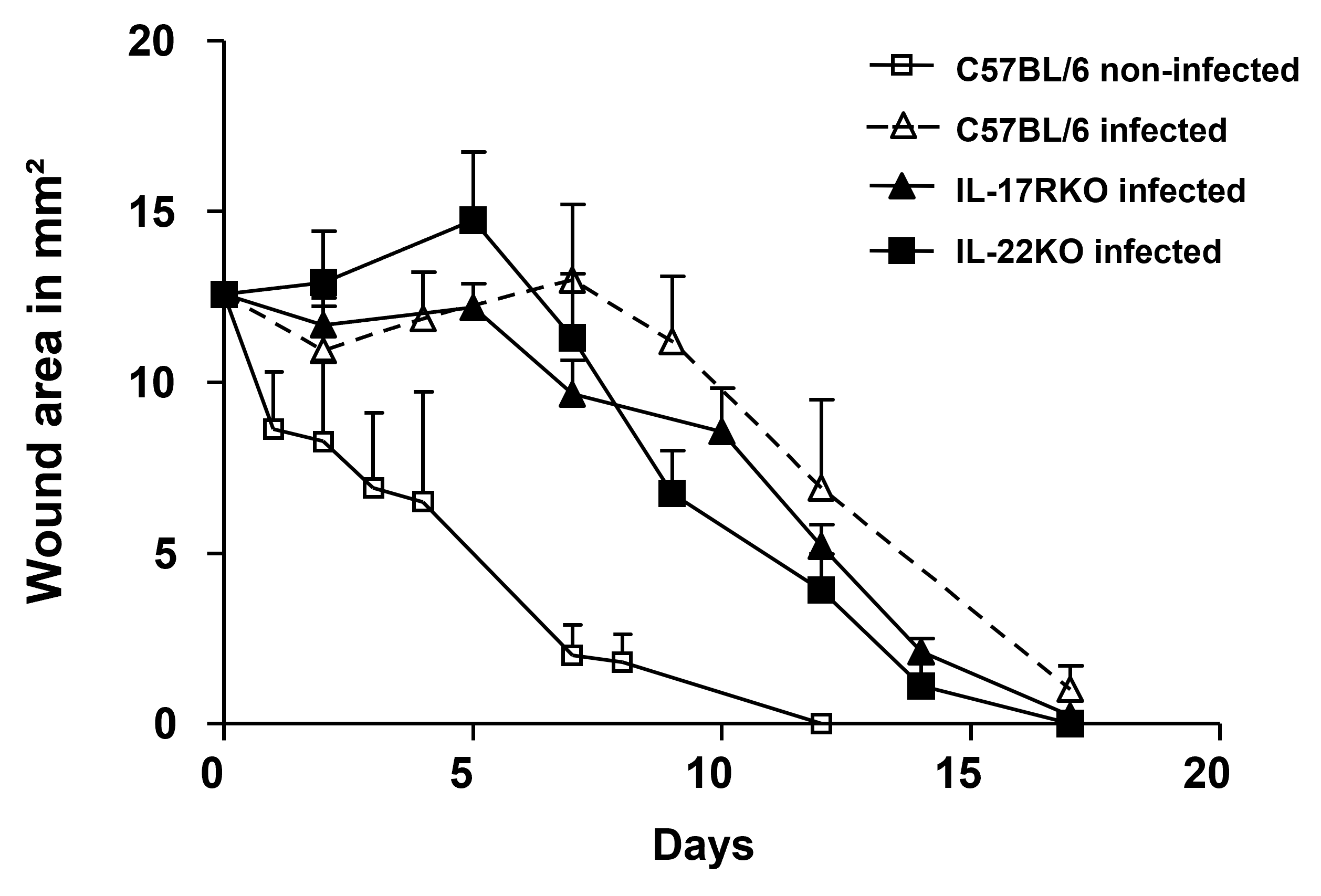


**Supplementary figure 1: Time course of wound closure in non-infected and infected control C57BL/6 mice and infected IL-22KO and IL-17RKO mice.** Wounds were infected at day 0 with a mixture of *S. aureus* and *P. aeruginosa* and measured wound area expressed in mm^2^ at indicated days. The data correspond to the mean +/- SEM of 2 independent experiments including 5 to 12 mice/group.


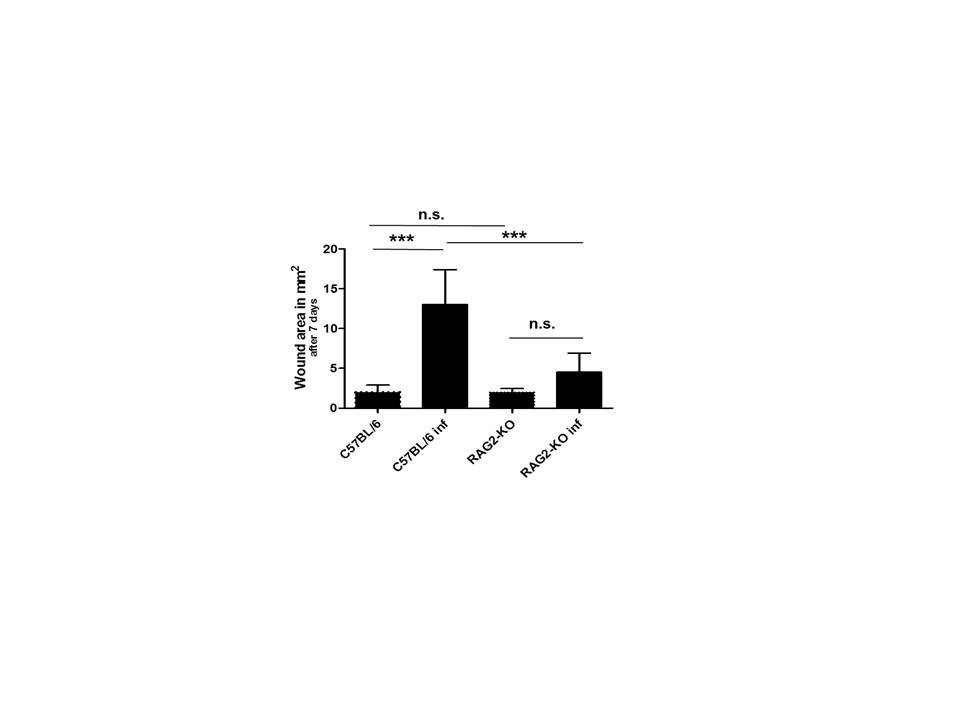


**Supplementary figure 2: Wound area measurement (in mm2) in non-infected and infected skin excisional wounds in control C56BL/6 and Rag2KO mice at day 7. The data correspond to the mean +/- SEM of 3 independent experiments; ***p<0.001.**
